# Outsourcing in Shiraz University of Medical Sciences; a before and after study

**DOI:** 10.1186/s42506-019-0010-0

**Published:** 2019-02-27

**Authors:** Omid Barati, Maryam Najibi, Ali Reza Yusefi, Hajar Dehghan, Sajad Delavari

**Affiliations:** 1 Health Human Resources Research Center, School of Management and Medical Informatics, Shiraz University of Medical Sciences, Shiraz, Iran; 2 Health Services Management, Student Research Committee, Shiraz University of Medical Sciences, Shiraz, Iran; 3 Health Care Management and Informatics School, Almas Building, Alley 29, Qasrodasht Ave, Shiraz, Iran

**Keywords:** Outsourcing, Public sector, Private sector, Outsourced services, Public-private partnerships

## Abstract

**Background:**

Outsourcing is a kind of participation between public and private sector. This should be monitored and supervised to enhance the quality of outsourced services and to prevent new problems in this area. Shiraz University of Medical Sciences (SUMS) hospitals increasingly use outsourcing in recent years.

**Objectives:**

The present research aimed at comparing outsourced departments of SUMS from economic view, accessibility of services, and service quality during the years 2010–2012.

**Methods:**

A before and after descriptive and analytical design was applied in outsourced departments of SUMS in 2014. First, 17 indicators were extracted by Delphi technique. Then, all outsourced units were assessed using economic, access to services, and quality indicators during 2010 to 2012.

**Results:**

After outsourcing, in all pharmacies and dentistry units, except one, loss decreased and benefit increased from public sector viewpoint. The number of personnel for one pharmacy and two laboratories was decreased, while it remained unchanged for dentistry units. The total number of clients was increased for all pharmacies and laboratories and decreased for one dentistry unit. Patient satisfaction for pharmacies, laboratories, and dentistry units was 73.4%, 80.3%, and 78.5%, respectively. Also, employer’s satisfaction from contraction was 60%, 68%, and 93.3% for pharmacies, laboratories, and dentistry units, respectively.

**Conclusion:**

Outsourcing as an effective strategy resulted in increase in the personnel, client, and stakeholder satisfaction. Also, it increased benefit and decreased cost for public sector. It is recommended that rules for the implementation of this strategy and monitoring the private sector should be defined.

## Introduction

In recent decades, reform in health systems mainly focused on financing, resource allocation, service delivery, and equity. In all reforms, arrangement of public and private sector and balance between them is a critical issue [1]. Reform in service delivery was performed via a policy named decentralization which mainly aimed at improving efficiency and responsiveness [1]. Privatization is one of the main types of decentralization which could be defined as adaptation of public management with market rules. Accordingly, privatization is not just a change in institutional possession but it could change the management manner, goals, and incentives [2].

Outsourcing is one of the forms of public-private partnership (PPP). In fact, outsourcing is a purchasing mechanism in which an organization purchases a special service at an agreed quality and quantity for a determined period from a service provider which is out of the organization [3, 4] and controlled via a contract or collaborative management [5]. Outsourcing could merge private sector advantages—such as efficiency [1, 6] and consumer satisfaction [7, 8]—into the public sector and avoid its disadvantages—such as inattention to equity and social responsibility [9]. This could result in generating internal market or quasi-market in the public sector that promotes competition [10, 11]. Also, outsourcing could improve accessibility, equity, equality, and efficiency and meanwhile create an atmosphere for collaboration of the private sector with the public sector [3].

Studies on outsourcing support it as it improves productivity and quality and decreases the costs. For example, in Greece, Maschuris and Kondylis stated that outsourcing in public hospital could result in improvement in service quality and patient satisfaction [7]. Taiwanese public hospitals decreased the number of needed staff along with improvement in their productivity and morale [12]. Similarly, an Indian study indicated that public hospitals that use outsourcing could decrease direct and indirect costs by about 40% [13]. So, outsourcing in health market is growing in all developing countries as a type of reform [14] for improving effectiveness [15, 16] and efficiency [16].

Outsourcing could resolve several issues in health systems and hospitals that result in the development of private sector partnership. As Aksan et al. stated, private sector partnership in Turkey is growing during recent years [17]. Mayson et al. suggest advances in treatment technology, difference in accessibility of healthcare, and resource scarcity as reasons for private sector growth. Even in developed countries, outsourcing is a strategy for reducing the executive workload of government. Bellenghi et al. assert that about 80% of US hospitals devolve health information services to expert deliverer based on the directive of the Association of Health Information Outsourcing Services (AHIOS) [18]. Nevertheless, inappropriate management of outsourcing could hinder managers to achieve their goals and create some deficiencies [5].

In Iran, outsourcing is growing as stated in several upstream documents to improve service quality and patient satisfaction and to decrease healthcare costs [14, 19]. Thus, policymakers need to appraise outsourcing to understand its weaknesses and strengths and consequently make decisions for its improvement. Shiraz University of Medical Sciences has outsourced health services based on upstream regulations in IR Iran. It outsources a number of service delivery units annually, based on managers’ decisions using different types of contracts with the private sector. Since Iranian medical universities are in the beginning of outsourcing, they need to have a better understanding of its consequences and outcomes. Given the importance and wide range of outsourcing, it seems necessary to evaluate outsourcing in terms of its goal attainment and determining the suitable type of contract. The present research aimed at comparing outsourced departments of Shiraz University of Medical Sciences (SUMS) from economic view, accessibility of services, and service quality during the period of 2010 to 2012.

## Material and methods

### Types of outsourcing

Type of outsourcing in Shiraz University of Medical Sciences was mostly lease, management, and collaboration, any type of which has its own profits and loss based on the type of the service.

In the lease contract, the contractor rents the governmental medical center by paying some fees and operates the medical center; instead, the contractor has the right to collect the income. In this case, all the commercial risks are transferred to the contractor. The responsibility of capital costs would be on the governmental sector. In the management contract, the governmental sector pays the private organization to manage a medical center unit and the government offers all the services needed as well. In this type, the decisions for hiring medical and health specialist workforce and procurement and purchasing of the medication and medical supplies are done by the government. However, the responsibility of the capital costs and commercial risks still remains on the government. In collaborative outsourcing, the profits are divided between the private sector and government based on their agreement [20].

Collaboration between governmental and private sectors in the form of contract could bring about potential risks. For instance, the presence of the private sector next to the governmental sector in an unorganized and uncoordinated pattern can cause cost pressure and overload on the governmental sector. Consideration of the determined framework and content could be an important starting point in creating coordination and solidarity in the process of contracting. Also, identifying the type of contract between governmental and private sectors in the health sector, type of payment in these contracts, method of monitoring, and supervision on contracts will have a potential effect on the contracts which they should specify in the contents of the contract precisely. In this case, there will not be any ambiguity and uncertainty for the parties of the contract. In addition, it could be used as guidance and instruction which help to improve the relationship between governmental and private sectors in the health field [21].

### Study design and data collection

A before and after descriptive design was applied in outsourced departments of SUMS. All outsourced departments which comprised of five pharmacies, five laboratories, and three dentistry departments during 2010 to 2012 were surveyed. First, a review of studies about outsourcing was conducted and 17 indices were identified for outsourcing assessment. Afterward, these indices were evaluated using the Delphi method in two rounds. Study population was experts who have sufficient knowledge about outsourcing to finalize indices. Experts were members of outsourcing workgroup of SUMS, hospital managers, deputies of treatment and logistics of SUMS, and professors and researchers in the field of healthcare and hospital management. Inclusion criteria were having experience of more than 1 year, having related education in the field of health management, and having sufficient information and knowledge about outsourcing. Based on inclusion criteria, 30 experts were selected using purposive sampling and were informed about the Delphi objectives and methods. Finally, 25 experts agreed to collaborate in the study.

A Delphi questionnaire for prioritizing criteria of outsourcing evaluation was administered among experts for the first round. The questionnaire was closed response with three choices including “agree,” “vague,” and “disagree” for each question. At the first round, data were analyzed and agreements below 30% were ignored, between 31% and 70% were entered into the second round, and more than 71% were confirmed. Then, the second round of Delphi was done with criteria based on three choices of the first round. Finally, 10 criteria were selected for outsourcing evaluation which was categorized into three domains including economic, accessibility, and service quality. Economic criteria were investment expenditures, current costs, salary and compensation costs, overhead costs (water, electricity, and gas), revenue, profit, and loss before and after outsourcing. Accessibility criteria were the number of personnel, number of clients, and activity hours. Service quality criteria included patients’ satisfaction and employers’ satisfaction from the contractor. After finalizing the criteria, a form was designed for gathering data about criteria before and after outsourcing based on available documents.

For measuring patient satisfaction in outsourced departments, a survey was done on patients who are referred to outsourced departments for getting services. Based on sampling formula, a sample of 384 was selected for each department with 0.95 degree of accuracy. Accordingly, a sample of 1152 was selected using stratified sampling based on share of pharmacy, laboratories, and dentistry departments.

Patient satisfaction questionnaire included 15 closed-answer questions with Likert choices “totally agree,” “agree,” “no difference,” “disagree,” and “totally disagree”. Questionnaire validity was approved using expert opinions, and reliability was tested with Cronbach’s alpha which was calculated as 0.85. Since satisfaction before outsourcing had not been measured, satisfaction comparison was not possible. Also, because of heterogeneity issues, we could not compare outsource departments with public ones. Thus, the comparison was not done and only satisfaction after outsourcing was analyzed. For measuring employer (SUMS) satisfaction, a checklist was used and filled based on monthly assessments by SUMS inspectors.

## Statistical analysis

Data were analyzed using descriptive statistics via MS Excel and SPSS software version 13 (SPSS Inc., Chicago, IL, USA).

Profit/loss change percentage is calculated based on the following formula:

$$ \mathrm{Profit}/\mathrm{loss}\ \mathrm{change}\ \mathrm{percentage}=\frac{\mathrm{Profit}/\mathrm{loss}\ \mathrm{after}\ \mathrm{outsourcing}-\mathrm{Profit}/\mathrm{loss}\ \mathrm{before}\ \mathrm{outsourcing}}{\mathrm{Profit}/\mathrm{loss}\ \mathrm{before}\ \mathrm{outsourcing}}\times 100 $$

## Results

During 2010 to 2012, 13 medical units in Shiraz University of Medical Science were outsourced to private sector, among which, 67.9% were leased including 5 pharmacies, 1 dentistry, and 3 lab units; 15.4% were collaborative including 2 dentistry units and 1 physiotherapy units; and 7.7% were management type including 2 lab units (Table 1).

**Table 1:** The outsourced medical units in Shiraz University of Medical Sciences, Iran, during 2010–2012

Unit name	Number of beds	Bed occupancy rate (%)	Date of outsourcing	Type of outsourcing	Cost of contract/Yearly US dollars
Zeinabieh Hospital’s pharmacy	196	68.5	20/04/2012	Leased	30,695.44
Lamerd Hospital’s pharmacy	102	81.3	23/02/2011	Leased	3549.16
Ali Asghr Hospital’s pharmacy	117	78.68	25/09/2011	Leased	21,902.47
Hafez Hospital’s pharmacy	165	89	20/01/2013	Leased	23,021.58
Emam Bagher Ghir va Karzin Hospital’s pharmacy	22	75	8/04/2010	Leased	1758.59
Khonj Medical Center Dentistry	–	–	28/02/2013	Collaborative (20% governmental and 80% private)	2097.52 (+) 20% of university’s share
Bigherd Medical Center Dentistry	–	–	28/02/2013	Collaborative (20% governmental and 80% private)	1438.84 (+) 20% of university’s share
Sede Eghlid Medical Center Dentistry	–	–	3/30/2012	Leased	2877.69 (+)
Eb-e-Sina Hospital’s lab	229	95	28/01/2012	Leased	7050.35
Shooshtari Hospital’s lab	38	94.7	21/01/2012	Leased	3490.54 (+)
Ghotb aldin Hospital’s lab	66	69.6	20/02/2011	Leased	5915.26 (−)
Lamerd Hospital’s lab	102	81.3	20/02/2011	Leased	1918.46 (+)
Dabiran Medical Center’s lab	–	–	22/11/2011	Management	0^*^

*In Dabiran Medical Center’s lab, capita rural insurance has been paid as service delivery to the contractor; thus, the total income and profits of university was zero

The highest cost and profits in terms of economic indicators belong to Zeinabie Hospital’s pharmacy with 1812% profit, and the least costs and profits in terms of economic indicators belong to Ali Asghar Hospital’s pharmacy with − 78%. Moreover, in terms of access indicators, Hafez Hospital gained maximum percentage of personnel changes with 175% growth in the number of staff and Ali Asghar Hospital’s pharmacy gained minimum percentage of personnel changes. As to the customers, except Ghir Karzin Hospital’s pharmacy, the remaining pharmacies experienced increased amounts of customers after outsourcing; the highest growth in terms of customers belonged to Lamerd Hospital’s pharmacy.

In terms of indicators of the quality of service, the satisfaction of patients was good in 60% of pharmacies, while 40% were evaluated as moderate. Furthermore, based on the committee of reduction tenure report of Shiraz University of Medical Sciences, the employer satisfaction of the contractor’s performance was moderate, good, and weak in 60%, 20%, and 20% of pharmacies, respectively (Table 2).

**Table 2:** Comparison of the performance of outsourced pharmacies in Shiraz University of Medical Sciences before and after outsourcing, Iran, 2010–2012

Name of the unit and type of outsourcing		Zeinabieh Hospital’s pharmacy		Lamerd Hospital’s pharmacy		Ali Asghr Hospital’s pharmacy		Hafez Hospital’s pharmacy		Emam Bagher Ghir va Karzin Hospital’s pharmacy	
Indicator		Leased (before/after outsourcing)		Leased (before/after outsourcing)		Leased (before/after outsourcing)		Leased (before/after outsourcing)		Leased (before/after outsourcing)	
		Before	After	Before	After	Before	After	Before	After	Before	After
Economic indicators	Expenses*	266,986.41	1145.75	94,054.09	532.90	460,964.56	852.65	404,476.41	1598.72	40,873.96	159.87
	Income*	268,531.84	30,695.44	47,961.63	3543.83	559,552.35	21,902.47	420,836.66	23,021.58	39,621.63	1758.59
	Profit/loss*	1545.43	29,549.69	− 46,092.5	3010.93	98,587.79	21,049.82	16,360.25	21,422.86	− 1252.33	1598.72
	Percentage in profit and loss changes	1812		106		− 78		30		227	
Access indicators	Number of staffs	5	12	3	7	6	5	4	11	3	6
	Number of clients	80,244	150,492	36,000	43,696	63,000	98,000	180,564	212,436	4992	3000
	Working hours of unit	24 h	24 h	24 h	24 h	24 h	24 h	24 h	24 h	24 h	24 h
	Patient satisfaction	N/A	80%	N/A	80%	N/A	60%	N/A	83.6%	N/A	63.4%
	Employer’s satisfaction from contractor	N/A	60%	N/A	80%	N/A	40%	N/A	60%	N/A	60%

*Currency numbers are calculated based on US dollars

In the outsourced labs, in terms of economic indicator, the highest percentage change in profit and loss belonged to Lamerd Hospital’s lab with 214% profit and the lowest percentage change in profit and loss belonged to Ghotb Aldin Hospital’s lab with 76% profit; the income of the university was zero in the labs with management type of outsourcing; in this way, all the income from Ghotb Aldin Hospital’s lab was allocated to the private sector, and in Dabiran Medical Center’s lab, capita rural insurance has been paid as service delivery to the contractor; thus, the total income and profits of the university was zero.

Also, in terms of access indicators, Ebn-e-Sina Hospital had the highest growth in the number of staff and Ghotb Aldin Hospital, with a decrease of staff from 11 to 8 persons, had the lowest growth in the number of staff. In terms of the client’s referral to the labs, there were an increased number of clients after outsourcing for all labs; the highest amount belonged to Shooshtari Hospital’s lab.

In terms of service quality indicators, the patient’s satisfaction with the performance of 80% of labs was evaluated as good and perfect while 20% of the labs were evaluated as moderate. Moreover, based on the committee of reduction tenure report of Shiraz University of Medical Sciences, the satisfaction of the employer from the contractor’s performance in 60% of the units was evaluated as good, 20% as moderate, and 20% as weak (Table 3).

**Table 3:** Comparison of the lab’s performance in Shiraz University of Medical Sciences before and after the outsourcing, Iran, 2010–2012

Name of the unit and type		Ebn e Sina Hospital’s lab		Shooshtari Hospital’s lab		Ghotb aldin Hospital’s lab		Lamerd Hospital’s lab		Dabiran Medical Center’s lab	
Indicator		Leased (before/after outsourcing)		Leased (before/after outsourcing)		Management (before/after outsourcing)		Leased (before/after outsourcing)		Leased (before/after outsourcing)	
		Before	After	Before	After	Before	After	Before	After	Before	After
Economic indicators	Expenses*	70,770.05	293.09	33,120.17	266.45	34,532.37	6794.56	16,573.40	239.80	3916.86	0
	Income*	34,905.40	7061.01	27,045.03	3490.54	5755.39	0	15,107.91	1918.46	4796.16	0
	Profit/loss*	− 35,864.7	6767.92	− 6075.14	3224.09	− 28,777	− 6794.56	− 1465.49	1678.66	879.3	0
	Percentage in profit and loss changes	118		153		76		214		− 100	
Access indicators	Number of staffs	3	6	9	6	11	8	3	5	1	2
	Number of clients	8172	12,924	22,356	38,568	12,000	15,492	21,888	25,476	4200	7128
	Working hours of unit	24 h	24 h	24 h	24 h	24 h	24 h	24 h	24 h	Morning	Morning
	Patient satisfaction	N/A	94.3%	N/A	60%	N/A	84.4%	N/A	76%	N/A	86.6%
	Employer’s satisfaction from contractor	N/A	80%	N/A	40%	N/A	80%	N/A	80%	N/A	60%

*Currency numbers are calculated based on US dollars

In terms of economic indicators in the outsourced dentistry units, the highest percentage of change in profit and loss belonged to Khonj Medical Center’s dentistry units with 798% profit, and the lowest percentage of change in profit and loss belonged to Sede Eghlid dentistry unit with − 60% of loss.

In terms of access indicators, there was no change in the number of staff in Sede Eghlid dentistry unit and also the number of clients decreased after outsourcing.

In terms of the employer satisfaction (Shiraz University of Medical Sciences) from the contractor, the results showed that the lowest grade belonged to Sede Eghlid dentistry unit, and in two other units, it was evaluated as perfect. Besides, in terms of patient satisfaction, there were no major changes between the units (Table 4).

**Table 4:** Comparison of the dentistry unit’s performance in Shiraz University of Medical Sciences before and after the outsourcing process, Iran, 2010–2012

Name of the unit and type		Khonj Medical Center’s dentistry		Bigherd Medical Center’s dentistry		Sede Eghlid Medical Center’s dentistry	
Indicator		Collaborative (20% government–80% private)		Collaborative (20% government–80% private)		Leased	
		Before outsourcing	After outsourcing	Before outsourcing	After outsourcing	Before outsourcing	After outsourcing
Economic indicators	Expenses*	3836.93	191.84	3836.93	159.87	11,191.04	319.74
	Income*	3517.18	2104.98	3277.37	1438.84	17,585.93	2877.69
	Profit/loss*	− 319.75	1913.14	− 559.56	1278.97	6394.89	2557.95
	Percentage in profit and loss changes	798		328		− 60	
Access indicators	Number of staffs	1	1	1	1	2	2
	Number of clients	1608	3120	1632	3264	1500	700
	Working hours of unit	Morning and afternoon	Morning and afternoon	Morning and afternoon	Morning and afternoon	Morning and afternoon	Morning and afternoon
	Patient satisfaction	N/A	86.8%	N/A	86%	N/A	80.6%
	Employer’s satisfaction from contractor	N/A	100%	N/A	100%	N/A	80%

*Currency are calculated based on US dollars

## Discussion

Comparison of the situation of the outsourced units of Shiraz University of Medical Sciences before and after the outsourcing process revealed that the type of outsourcing in this university was mostly lease, management, and collaboration, any type of which has its own profits and loss based on the type of the service.

The results also revealed that outsourcing of health services based on leasing method is a kind of successful strategy for pharmacies because, in most of the pharmacy units, we experienced the growth of profit and also improvement of access indicator. Tourani et al. found that pharmacy outsourcing at Firoozgar Hospital in Tehran had resulted in cost saving in personnel, medication, and leasing costs. Besides, the number of personnel (as an index for accessibility) was increased from 9 to 14 while their educational level and the number of performed prescriptions were increased. Moreover, the time spent by the manager for managing pharmacy affairs was decreased [3]. A research in the USA showed that outsourcing resulted in decrease in pharmacy cost and number of personnel that saved $59,000 in the first year [22]. As all these study shows, pharmacy costs were decreased after outsourcing which is corresponding with current findings.

In the current research, the only exception from decreasing pharmacy cost was Ali Asghar Hospital’s pharmacy. Studies showed that the governmental sector is lost due to inaccurate expertise rent cost and selection of inappropriate contractor. Due to pharmaceutical sanctions, the contractor is delayed in paying the insurance claims from the insurance companies and is faced with lack of liquidity and lack of supply of the medicine, which finally caused the employer’s dissatisfaction and cancelation of the contract for the next year. Based on Maschuris and Kondylis research in Greece, some factors such as cost of contract (suggested price), quality of services, diversity and range of services, past history, financial state, and also contractor’s reputation should be considered in choosing the contractor by the medical units to fulfill outsourcing goals such as cost savings, patients’ satisfaction, resolution of the lack of fund and staff, and management focus on core activities [7].

With respect to the service quality indicator in the present study, patient satisfaction, most of the units were in a desirable level and the employer satisfaction from the contractor’s performance was reported in a moderate level.

The study also showed that 40% of the outsourced pharmacies were moderate regarding the quality of service indicators. Mohaghegh et al. revealed that the pharmacies’ outsourcing approach including increase of patient satisfaction may fail even though it promised care improvements; thus, the measures such as clear and comprehensive contracts should be taken, and practical mechanisms such as monitoring and evaluation should be accomplished [23]. One of the factors that could hinder improving customer satisfaction due to outsourcing is the limited number of private suppliers and hence lack of competitive market [24]. Therefore, by considering patient satisfaction in evaluating the outsourced unit performance, it is suggested that in the provision of the contract, patient satisfaction has to be defined as the contractor’s controlling tool, and in the case of low satisfaction, penalty system will be specified, and by emphasizing on performance contract, payment mechanism will be modified in a way that the payments to private sectors in addition to prescription cost will be related to customer’s satisfaction.

In the present research, the outsourced labs had a loss before outsourcing due to low tariffs of lab services, the high cost of consuming materials, and high annual personnel costs. Therefore, the university has used the lease or management outsourcing strategy for outsourcing. In comparison of these two types of outsourcing, the lease outsourcing was more successful because the increase in its profitability was remarkably higher than management outsourcing. Some factors including staff’s low income and benefits compared with the governmental sector, longer working hours, diversity in services, saving raw materials, and patients’ marketing strategy have caused profit for the private sector. Also, in this outsourcing, besides an increase of clients and a decrease in costs, a significant growth was seen in the profit of the private sector.

Omrani et al. showed that after labs’ outsourcing, laboratories income increased 51% due to more customers, longer working hours, not referring to other labs, and diversity in the lab’s experiments [25]. Another study in Iran showed the positive effect of signing a contract in lab leasing to the private sector by comparing income and expenses of these units before and after the contract [26].

In the management outsourcing of Ghotb Aldin Hospital’s lab, although the goals of outsourcing such as cost saving and prevention of loss have been fulfilled, it is possible to convert this unit to a profitable one by using some solutions such as increase in the service diversity, patient marketing, and leasing outsourcing. Also, in management outsourcing, Dabiran Medical Center’s lab was profitable for the university but it was outsourced to the private sector to improve service access and quality indicators as well as governmental sector obligation to decrease tenure. In this regard, rural per capita amount of insurance was paid to the private sector and the contractor was obliged to deliver high-quality services, attract maximum clients, and pay current fees of the lab. In management outsourcing, even though there were no financial benefits for the government, an improvement in the access to service indicators by using two non-governmental workforces, quick response, and more client attraction was experienced.

In terms of quality of service indicators, the satisfaction indicators were in a desirable level in the outsourced labs. The research in Iran by Omrani et al. showed that responsibility and behavior of the lab’s personnel improved after outsourcing [25]. In order to improve patient and personnel satisfaction, it is suggested to hire specialized workforce and try to decrease medical errors. A study in Arizona revealed that the medical error increased after lab outsourcing. The number of misdiagnosis increased, causing physical and financial problems to the patients due to the use of unprofessional workforce in the labs [27]. Based on the abovementioned results, it is recommended that if the patients’ satisfaction and the service quality are low in the outsourced labs, it is possible to decrease the contractor’s payment as a fine solution.

Findings indicate that from one and three dentistry units that were outsourced by leasing and partnership, respectively, profitability was increased in two partnership outsourcing units (Khonj and Bigherd). Besides, in these two units, accessibility and number of patients were increased and the satisfaction of SUMS and patients’ satisfaction from delivered services were high. In the lease outsourcing of Sede Eghlid Dentistry Center, this unit was profitable for the university before outsourcing. Due to the placement of this unit in a deprived area, the obligation to decrease government tenure, and the absence of appropriate contractor, the university outsourced the unit as a lease unit in order to prevent closure and improve access to service indicators. The condition of this unit in terms of patient satisfaction and employee after outsourcing was in a desirable level.

Finally, the results of the study showed that in most of the outsourced units with the increase in the number of staff in private sector and with the increase in the number of clients, we experienced increase in profitability and reduced costs for the governmental sector; therefore, outsourcing for medical units can be an effective strategy. As several studies revealed, if outsourcing strategy is done by risk and cost assessment along with careful and measured approach, it can be an effective strategy resulting in benefits for management, staff, contractors, patients, and even hospitals [28–30]. Moreover, it is suggested that hospitals, especially the governmental ones, can use outsourcing benefits as a strategy for a full-time service and reduction of the human resource constraints, more manager’s effort to hospital management, time-saving, productivity improvement, and staff morale. Also, an outsourcing strategy can be used as an approach to help the hospitals to attract new sources without paying any costs [12].

## Conclusion and recommendations

Outsourcing in SUMS curative units has increased benefit, accessibility indicators, and service quality. Hence, outsourcing could be suggested as a reform mechanism in the health system. Moreover, defining indicators for evaluation of outsourcing and continuous monitoring of indicators are highly recommended for better analysis by policymakers.
